# Electrochemical Behaviour of the Antioxidant Gallic Acid Using a Low-Cost Screen-Printed Carbon Sensor and Its Exploitation for Banana Wine Analysis

**DOI:** 10.3390/foods14234070

**Published:** 2025-11-27

**Authors:** Sotirios I. Ekonomou, Olena Doran, Adrian Crew, John P. Hart

**Affiliations:** Centre for Sustainable Agri-Food and Environment (SAFE), Faculty of Health and Applied Sciences, University of the West of England, Bristol BS16 1QY, UK; sotirios.oikonomou@uwe.ac.uk (S.I.E.); olena.doran@uwe.ac.uk (O.D.); adrian.crew@uwe.ac.uk (A.C.)

**Keywords:** banana wine, gallic acid, antioxidants, electrochemical sensors, cyclic voltammetry

## Abstract

A low-cost screen-printed carbon electrode (SPCE) was used to determine gallic acid (GA) in banana wine (BW). Cyclic voltammetry (CV) showed GA oxidation in Phosphate-Buffered Saline (PBS, pH 7.0) with 10% ethanol was diffusion-controlled, forming a quinone species. This supporting electrolyte was applied for calibration using the identified CV and differential-pulsed voltammetry (DPV) peaks, whose low potentials ensured good selectivity and stability. Linearity was obtained between 0.25–5.00 μM GA, suitable for BW analysis. BW was filtered, diluted (1:20) in electrolyte, and analysed via the standard addition method. GA concentrations were 7.369 μM (CV) and 7.570 μM (DPV), with no significant differences. Further validation of the voltammetric procedure using fortified BW confirmed its reliability, with excellent recoveries of 94.41% (CV) and 99.33% (DPV). The SPCE-based voltammetric approach offers a simple, accurate, and low-cost method for GA determination in BW, combining good sensitivity, selectivity, and reproducibility.

## 1. Introduction

Bananas are one of the world’s most produced, traded, and consumed fruits, with over 1000 varieties. The popular Cavendish banana makes up nearly half of global production, estimated at 50 million tonnes annually. In low-income, food-deficient countries, bananas are vital for household food security and income as a cash crop [1]. Many of these are consumed fresh, but large quantities are often wasted during peak harvest periods due to factors such as increased temperatures, fluctuations in humidity, inadequate storage conditions, improper handling practices, improper transportation, and microbial contamination. Additionally, banana skin accounts for about 35–40% of the total fruit weight, with more than 36 million tonnes of banana skin generated annually [2,3,4]. During processing, banana skins are considered by-products or waste, which pose serious environmental hazards and are typically discarded with general waste. Furthermore, imperfect green bananas are rejected as they cannot be traded, thereby increasing the waste generated in banana production [4,5]. Therefore, the utilisation of overripe bananas or surplus fruits for wine production has emerged as a viable and efficient strategy for waste valorisation and resource optimisation [2]. These banana agrifood wastes, although sometimes used as compost or soil conditioner, have low market value and could be utilised in producing high-value banana wine (BW) using bananas and all their by-products. Therefore, the utilisation of overripe bananas or surplus fruits for wine production has emerged as a viable and efficient strategy for waste valorisation and resource optimisation, offering a high-value use for both the fruits and their by-products [6].

Wine, one of the oldest known fermented beverages, is a vital component of regional culture and a significant market segment in the agribusiness industry [7]. Fruit wines are gaining more recognition due to the diverse consumer preferences and demands, which increasingly prioritise climate change, sustainability, and health concerns. In this context, winemaking can enhance the value of fruits for consumption and address the issue of fruit waste during production and marketing, offering significant development potential for the fruit wine industry and economic benefits for localised production in low-income countries [8].

Utilising banana peels in wine making can improve the physicochemical characteristics of wine and release its phenolic and bioactive compounds, which are bound to insoluble plant compounds, increasing their availability for absorption during consumption [9]. Banana peels are rich in phenolic and bioactive compounds, known for their anticancer properties [10], antibacterial [11] and antioxidant capacities [12,13], as well as other health benefits [2]. Gallic acid (GA), a naturally occurring triphenolic (3,4,5-trihydroxybenzoic acid) compound, is well known for its antioxidant and anti-inflammatory properties and is one of the main phenolic compounds in bananas and banana peels [2,13]. Moreover, GA in wine is an important quality indicator, as its concentration can affect bitterness, colour, antioxidant properties, and overall taste, thereby influencing consumer acceptance [14].

The content of GA in different samples, such as fruits, nuts, beverages and other extracts can be determined by chromatographic techniques, including Gas Chromatography–Mass Spectrometry (GC-MS), high-performance liquid chromatography (HPLC), thin-layer chromatography Fourier-transform infrared spectroscopy (TLC FT-IR) [15], spectrometry, and other techniques such as Chemiluminescence analyses [16], differential scanning calorimetry (DSC) and thermogravimetry (TG) [17]. While these techniques are highly reliable and sensitive, they also have some drawbacks. These are costly instruments that require high levels of technical skill to operate and maintain. They employ complex methods of analysis, which involve pretreatment steps, and are non-environmentally friendly due to their time-consuming nature, and the high usage of analytes given the multiple steps involved [18,19]. On the other hand, screen-printed carbon electrode (SPCE) technology offers an attractive alternative due to its advantageous attributes and could be an excellent alternative and sustainable technique. They can be easily mass-produced in a wide range of geometries, and since carbon (used in the current study) is inexpensive, they can be manufactured economically and require only a few seconds or minutes for analysis, with minimal portions of analytes. Therefore, they can be considered disposable, and this leads to rapid methods of analysis [20,21].

To the best of the authors’ knowledge, this is the first study presenting the development of a simple method for detecting and quantifying GA in BW using a rapid, low-cost, and straightforward procedure based on plain SPCE sensors. This sensor would possess important attributes, including mass production at a low cost and the capability for rapid measurements by simply using small aliquots of liquid samples with minimal preparation. Both voltammetric techniques of Cyclic Voltammetry (CV) and Differential-Pulsed Voltammetry (DPV) used are very suitable for detecting low concentrations of GA in complex matrices and are both easy to perform and to interpret. This approach would alleviate the need for expensive laboratory techniques and help achieve the portability of an analytical sensor system, enabling small local producers to test GA levels during wine production on-site. This paper describes the steps involved in optimising the sensor, as well as its application to measuring GA in BW.

## 2. Materials and Methods

### 2.1. Instrumentation and Apparatus

All voltammetric measurements were conducted using an Emstat Blue potentiostat (PalmSens, Houten, The Netherlands), which was connected to a PC for data acquisition with PS Trace Software version 5.10.

Plain SPCE sensors were supplied by Gwent Electronic Materials Ltd. (Newport, UK). The working electrode for plain SPCEs was fabricated using a carbon ink (C2030519P4), and the reference electrode was fabricated using Ag/AgCl ink (C2130809D5). The working electrode area (3 × 3 mm) was defined using electrical insulation tape. A dual-glass electrochemical cell with an internal volume of 30 mL was used for all analytical measurements (Metrohm UK Ltd., Runcorn, UK). The electrochemical cell was temperature-controlled by flowing water from a connected water bath. Each sensor was used once and then discarded, so a fresh electrode was used for each measurement.

All pH measurements were performed using a Testo 205 (Testo Limited, Alton, Hampshire, UK) pH meter. Solutions were stirred using a magnetic stirrer (IKA, Tunbridge Wells, UK) and warmed at 30 °C using a HAAKE P5 water bath (Thermo Scientific, Loughborough, UK).

### 2.2. Chemicals and Reagents

All chemicals were obtained from Sigma Aldrich (Dorset, UK). Stock solutions of monosodium, disodium, and trisodium orthophosphate were prepared at a concentration of 0.2 M by dissolving the appropriate mass in deionised water, then titrated to the desired pH and diluted in the cell to achieve a working concentration of 0.1 M [18]. Sodium chloride was prepared at a 1.0 M concentration by dissolving the appropriate mass in deionised water, then diluted in the cell to achieve a final concentration of 0.1 M.

### 2.3. Banana Wine Preparation

Fresh, ripe organic bananas were obtained from a commercial food wholesaler in Bristol, UK, with the same expiration date and stored at room temperature. All bananas purchased were undamaged and at peak ripeness; all bananas were processed on the same day. Organic bananas with their skin were washed and weighed and then sliced into pieces of approximately 2 cm in width and placed in a stainless-steel pot. Tap water was added until just covering the bananas, which were then cooked at 95 °C and stirred regularly with a clean stainless-steel spoon until soft. The banana pulp was subsequently strained to separate the juice from the solid particles using a large muslin juicing bag in a manual crossbeam stainless-steel wine press. In total, 3.5 kg of food-grade sugar, 30 mL of lemon juice, and 5 g of Lalvin K1-V1116 yeast (Lallemand Brewing, Napa, CA, USA) were added to every 10 L of banana juice, which was subsequently fermented in 4 L sterile glass demijohns until completion (approximately 12 days) in the dark at an ambient temperature of 21 °C. A consistent juice extraction process ensured a uniform fermentation [22]. Each fermentation was split between two 2 L sterile glass bottles and stored at 4 °C.

### 2.4. Voltammetric Characterisation Procedure of Gallic Acid Using Different pH Values

To deduce the effect of pH on the oxidation behaviour of GA, CV was performed using the plain SPCE sensor from an initial potential of −0.1 V to a switching potential of +1.2 V vs. Ag/AgCl, with current measurements recorded at different scan rates of 20, 50, 100, and 150 mV s^−1^. DPV was also used at the same potential window (−0.1 V to +1.2 V vs. Ag/AgCl) and pulse amplitude 20 mV, pulse width 50 ms, step height 10 mV, step width 0.4 s, and scan rate 25 mV s^−1^.

For the standard addition method, a 1:20 dilution of the sample was prepared by adding 0.5 mL BW to 9.5 mL of 0.1 M, pH 7.0 phosphate-buffered saline (PBS). Standard additions of 0.25 to 0.75 μM GA were transferred to the electrochemical cell, and the solutions were kept at a constant temperature of 30 °C. A fresh, plain SPCE sensor was used to record the CVs and DPVs of each buffer solution in six replicates (*n* = 6). Blank solutions were tested as described, without the addition of BW and GA.

### 2.5. Calibration Procedure

Calibration studies were performed using GA solutions prepared in 0.1 M PBS at pH 7.0 over a concentration range of 0.25–5.00 μM. The electrochemical techniques employed were CV and DPV by potential cycling between −0.1 V to a switching potential of +1.2 V vs. Ag/AgCl. Aliquots of 25 μL of standards prepared in the same buffer solution were added to the cell solution, which was warmed at 30 °C. A fresh sensor was used to measure each concentration of GA. In the current study, we constructed calibration plots with the above standard solutions by taking current measurements at the peak potential of GA from the cyclic and differential-pulsed voltammograms. Since oxygen did not influence the GA oxidation or cathodic reduction, deaeration of the cell content was avoided. All CV and DPV runs for each concentration of test analyte were quantified using the method of standard addition. The Limit of Detection (LOD) was calculated as 3.3 times the standard error divided by the slope of the calibration plot between GA concentration and peak current.

### 2.6. Determination of Original Gallic Acid Concentration in Banana Wine and Recovery Study Using the Standard Addition Method

CV and DPV were next employed to analyse BW under the same operating conditions as in Section 2.5. An initial treatment of the BW sample was performed by filtration through a 0.20 μm Millipore filter (Merck, Darmstadt, Germany) to remove any potential interfering material present in the sample. Next, an aliquot of the filtrate was diluted in the voltammetric cell, using the buffer components mentioned earlier to obtain a final volume of 10.0 mL at pH 7.0. Both CV and DPV were then performed using the buffered wine sample with fresh SPCEs for these and all subsequent measurements. For the standard addition method, additions of a standard GA solution, prepared in the same supporting electrolyte, were made to the cell containing the filtered, diluted wine, resulting in added concentrations of 0.25, 0.50, and 0.75 μM; each of these solutions was analysed in turn. A total of five replicate analyses were performed for each solution, and all measurements were done at a temperature of 30 °C. Blank solutions were tested as described, without the addition of BW and GA.

For the recovery study, BW samples were initially placed into 5 mL Sterilin tubes (Thermo Scientific Z10PS, Leicestershire, UK) and used for recovery tests after a 1:20 dilution in the supported electrolyte as described earlier. A measured amount of GA standard stock solution was added to the samples to achieve a final concentration of 7.5 μM GA in the tube. Before analysis, the samples were filtered as described above. Every test was repeated five times (*n* = 5) using both CV and DPV.

### 2.7. Statistical Analysis

All data acquired were expressed as mean ± standard deviation (SD), and Microsoft Excel 365 (ver. 16.48) was used to plot graphs. Data were analysed using the paired two-sample *t*-test with IBM SPSS Statistics 26 software for macOS (SPSS Inc., Chicago, IL, USA) at a 5% level of significance.

## 3. Results and Discussion

### 3.1. Cyclic Voltammetric Characterisation of Gallic Acid Using Plain SPCE Sensors Under Acidic, Neutral and Alkaline Conditions

#### 3.1.1. Effect of pH on the Oxidation of Gallic Acid

**Figure 1:**
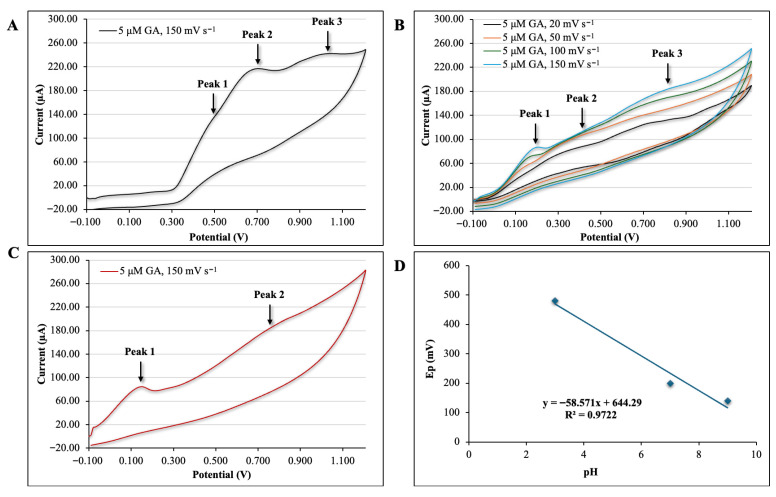
Cyclic voltammograms obtained with a plain SPCE sensor at (**A**) pH 3.0, scan rate: 150 mV s^−1^, (**B**) pH 7.0, scan rates: 20, 50, 100, and 150 mV s^−1^. (**C**) pH 9.0, scan rate: 150 mV s^−1^ in the presence of 5.0 μM of GA, and (**D**) plot of peak potential (E_p_) at pH 3.0, 7.0, and 9.0 and 150 mV s^−1^. All measurements were performed in 0.1 M phosphate-buffer solutions (pH 3.0, 7.0, and 9.0).

Initially, we investigated the effect of pH using cyclic voltammograms obtained in the presence of 5.0 µM GA dissolved in acidic (pH 3.0), neutral (pH 7.0), and alkaline (pH 9.0) supporting electrolytes, as shown in Figure 1A–C for plain SPCE sensors.

The CVs obtained in the presence of 5 µM GA dissolved in PBS, pH 3.0 (Figure 1A) exhibited three anodic peaks. An initial unresolved anodic peak at an inflection potential of Ep = +0.48 V (peak 1), adjacent to a more positive peak of Ep = +0.69 V (peak 2) and a more positive third anodic peak appeared at Ep = 1.00 V (peak 3) on the forward positive going scan, which resulted from the oxidation reactions of GA at the carbon working electrode. At this point, we considered that the responses probably resulted from the direct oxidation of the phenolic hydroxy groups, which is discussed further below. Cathodic peaks were absent on the reverse negative CV scans between −0.1 V to +1.2 V. This suggests that the overall reaction is irreversible in the range studied. A similar observation was made for acidic media using a reduced graphene oxide composite electrode, where no detectable reduction peak was observed, suggesting the irreversible electrochemical process of GA [23].

As shown in Figure 1B, when the supporting electrolyte was changed to PBS at pH 7.0, the CV anodic peaks shifted to more negative potentials compared to those found at pH 3.0. A well-defined peak, peak 1, was observed at Ep = +0.20 V, followed by two ill-defined peaks: Ep = +0.40 V (peak 2) and Ep = +0.80 V (peak 3). Therefore, the position of these anodic peaks is pH-dependent, indicating that the electrochemical oxidation reactions involve both H^+^ ions and electrons. There were no cathodic peaks on the reverse scan for gallic acid with this neutral buffer solution, indicating that the oxidation mechanism is consistent over this pH range in agreement with Gao et al. [23].

Finally, when the pH of the supporting electrolyte containing 5 μM of GA was changed to PBS at pH 9.0, the CVs shown in Figure 1C were obtained. As can be seen, the oxidation responses again shifted to more negative potentials, with peak 1 appearing at a potential of Ep = +0.12 V and an ill-defined peak 2 at approximately Ep = +0.75 V. This negative shift in the anodic responses implies that both H^+^ ions and electrons are involved in oxidation reactions.

Figure 1D shows the plot of E_p_ vs. pH for GA using the values deduced from Figure 1A–C, from which a slope of 58.57 mV/pH was measured. The theoretical slope of such a plot is as follows (1):

(1) $$\frac{E_{p}\;(V)}{pH}=59\;mV\;(\frac{m}{n})$$

where m is the number of protons and n is the number of electrons involved in the reaction. This implies that when the number of protons (m) and electrons (n) are equal, a slope of 59 mV will be measured. Since our slope value is close to this, we believe that this occurs in the present case [24].

#### 3.1.2. Mechanism of Gallic Acid Oxidation

**Scheme 1:**
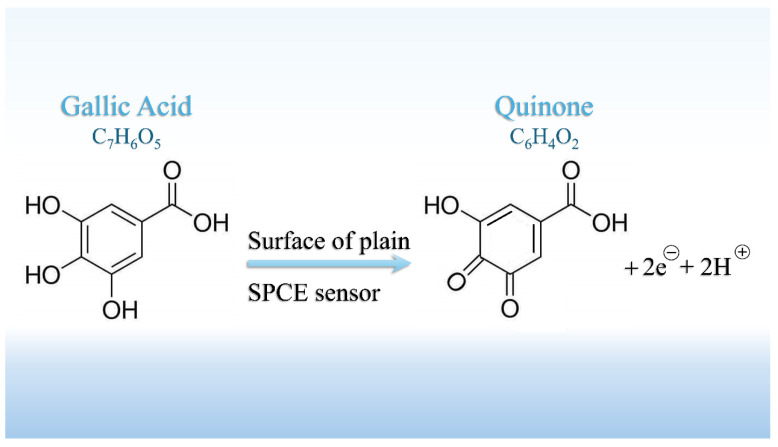
A schematic presentation of the gallic acid oxidation reaction on the surface of the plain SPCE sensor.

To deduce the number of electrons involved in the oxidation reactions mentioned above, we performed wave analysis of the cyclic voltammograms presented in Figure 1A–C. The αn_a_ value was calculated using the following relationship by Gilmartin et al. [25] (2):

(2) $${an}_{a}=\frac{48}{(E_{p}\;-{(E}_{p})/2)\;}mV$$

where E_p_ is the peak potential and E_p/2_ is the potential at half peak current. From this relationship, αn_a_ was deduced to be 1.2; as the value of α is usually close to 0.5, which implies that *n* = 2. This value is in agreement with that obtained for the oxidation of GA using a glassy carbon electrode [26]. Consequently, we may deduce that 2 protons are also involved in the oxidation reaction, which produced peak 1 of the cyclic voltammograms shown in Figure 1B. Therefore, we propose that the reaction shown in Scheme 1 occurs at the SPCE surface. It should be mentioned that the presence of the more positive peaks shown in Figure 1A–C may be the result of further oxidations occurring at the phenolic hydroxy species remaining on the quinone species obtained from the initial oxidation reaction. It is also suggested that the first oxidation processes, giving rise to the most negative CV peaks obtained with buffer solutions at pH 3.0 and pH 9.0, would also produce a quinone species.

#### 3.1.3. Effect of Scan Rates on Anodic Peak Current

**Figure 2:**
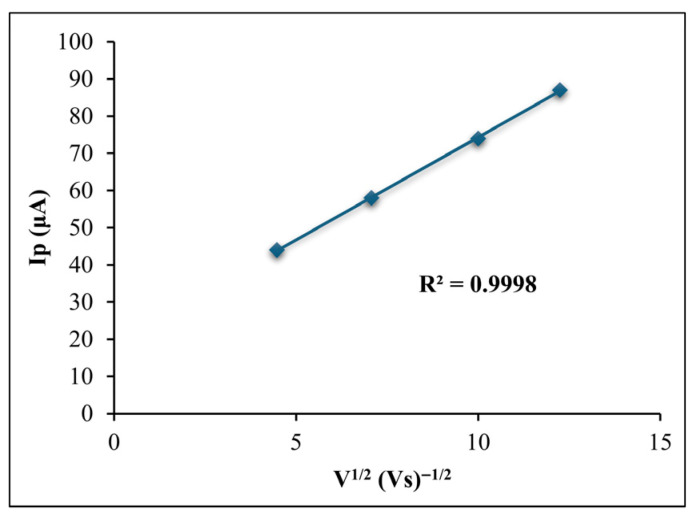
Plot of I_p_ vs. V^1/2^ the square root of scan rate for the anodic peak current in the presence of 5.0 μM of gallic acid. Each data point represents an experimentally measured anodic peak current at a scan rate of 150 mV s^−1^.

Next, we investigated the nature of the oxidation reaction by conducting a scan rate study at scan rates ranging from 20 to 150 mV s^−1^ at pH 7.0. As shown in Figure 2, there is a linear relationship between the magnitude of the peak current and the square root of the scan rate. This implies that the anodic reaction is diffusion-controlled under the solution conditions we employed in this study [27].

The results observed in Figure 1 and Figure 2 revealed that neutral conditions at pH 7.0, containing 10% (*v*/*v*) ethanol, would offer an appropriate supporting electrolyte for our later studies on wine analysis (Section 3.3). Therefore, Section 3.2 presents a calibration study to deduce the performance characteristics of the proposed voltammetric method.

### 3.2. Calibration Study of Gallic Acid

An initial study was required to establish the effect of GA concentration on the analytical response current. For this purpose, we selected a concentration range of 0.25 to 5.0 μM of GA, as this is suitable for wine analysis applications. As we have employed CV in the above characterisation studies, we first employed this technique for calibration purposes. DPV was used as an additional popular electrochemical technique, which has been shown to have some advantages for the analysis of complex matrices, such as fermented foods and beverages [28], red and white wine [29], biological fluids [30] and pharmaceutical samples [31].

The limits of detection (LOD) were calculated based on the standard error of the regression line (STEYX) of the current response and the slope of the calibration curve (S) according to the formula: LOD = 3.3 × (STEYX/S). The performance characteristics shown in Table 1 indicate that both voltammetric techniques possess the required linear range and LODs for the application to the measurement of GA in BW.

### 3.3. Analytical Application of the Plain SPCE Sensor to the Determination of Gallic Acid in Banana Wine

**Figure 3:**
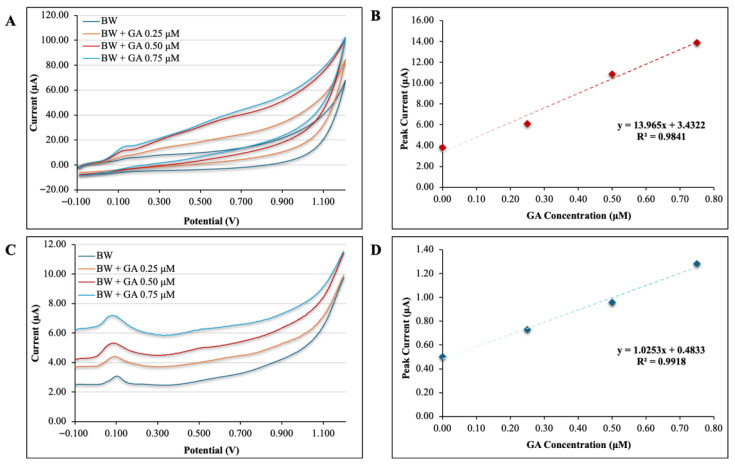
Voltammetric determination of gallic acid in banana wine with plain SPCE sensor using (**A**) CV, scan rate: 150 mV s^−1^, pH 7.0 and (**C**) DPV, scan rate: 25 mV s^−1^, pH 7.0. The standard addition curves for gallic acid in banana wine with (**B**) cyclic voltammetry and (**D**) differential-pulsed voltammetry.

For the analysis of BW, we used, as discussed earlier in the text, both the CV and DPV techniques in conjunction with the method of standard addition. The method of standard addition ensures that any effect of the sample matrix (BW) on the analytical response from the sample also affects the added standard of GA to the same extent, thereby limiting errors in the calibration process. Moreover, the use of both CV and DPV was employed as complementary techniques to establish the accuracy of the recovery data (Figure 3). Figure 3 shows representative CVs and DPVs with corresponding standard addition plots of GA, ranging from 0.25 to 0.75 μM. Clearly, the data in Figure 3A–D demonstrate that the proposed method is suitable for the determination of GA in BW.

To evaluate the proposed GA screen-printed voltammetric sensor for quantitative analysis, the device was applied to measure the antioxidant in BW. Prior to analysis, the wine was filtered, and an aliquot of the filtrate (0.50 mL) was added to an electrochemical cell containing a supporting electrolyte (9.50 mL). The sensor was then inserted into the diluted sample, and CV was performed using a scan rate of 150 mV s^−1^. The mean value obtained was 7.369 ± 0.194 μM (Table 2), corresponding to 1.254 ± 0.033 mg L^−1^, which is a similar concentration to that reported by other researchers, ranging from 0.30 to 2.90 mg L^−1^ banana peel extracts as determined by the reversed-phase HPLC method [13]. Li et al. [22] investigated the phenolic content of whole banana wine using HPLC, and the concentration of GA was 1.209 mg L^−1^. The above results demonstrate the reliability of our newly developed method, which is comparable to the results obtained using expensive and labour-intensive gold standard methods, such as HPLC. When DPV was employed, GA found in BW was equal to 7.570 ± 0.715 μM (1.287 ± 0.122 mg L^−1^), without statistically significant differences compared to CV (*p* > 0.05, Table 2). In addition, the reproducibility of CV and DPV, with RSDs of 2.6% and 9.6%, respectively (Table 2), indicates that reliable data can be expected when performing this type of analysis.

To further validate our new electrochemical approach for analysing GA in BW, an identical sample of the above wine was fortified with a known concentration of GA (7.50 µM) prior to filtration. The same measurement procedure was then performed on the filtrate. Table 3 shows the recoveries of added GA. The results in Table 3 indicate that the recoveries of 94.41% and 99.33%, as well as the RSDs of 2.90% and 2.04% for both developed CV and DPV techniques, respectively, involving our plain SPCE GA sensor, hold promise for the quality control analysis of BW.

A wide variety of sensors have been reported in the recent literature that can be employed for measuring GA in various food matrices and beverages, including black tea, pomegranate juice, and wines, as presented in Table 4. The examples shown in Table 4 demonstrate the reliability and effectiveness of using various electrochemical approaches for the measurement of low levels of GA in different food products. Although these have been successful for the type of food sample for which they were developed, they require a high degree of analytical skills and electrochemical principles, which will be problematic for small producers of wine and other products. In most cases, the sensors require additional preparation steps that involve the synthesis of specialised materials (e.g., activated pencil lead electrodes and NPs). For the purpose of rapid analysis in small companies and underdeveloped countries, a fully prepared, easy-to-use sensor would be highly desirable. Screen-printed technology for the production of simple, low-cost, disposable devices offers such advantages.

## 4. Conclusions

The present paper reports for the first time the feasibility of using disposable, low-cost screen-printed carbon sensors for determining the electrochemical behaviour of gallic acid. It was also demonstrated that the oxidation reactions can be applied for the quantitative analysis of gallic acid at the micromolar level. An advantage of this approach is that it requires only a simple pre-treatment step, involving the filtration of a representative wine sample (banana wine), followed by dilution with the supporting electrolyte. The analytical measurement step can be performed using either cyclic voltammetry or differential-pulsed voltammetry. Moreover, the identification of a neutral buffer solution (pH 7.0) as the supporting electrolyte resulted in a very low oxidation potential of the working electrode (+0.20 V), which is considerably lower than reported methods which employ acidic electrolytes. The advantage of the low anodic peak potential is that this is expected to result in significantly improved selectivity. In addition, this approach eliminates the possibility of any memory effect on the electrode response because each electrode is electrochemically active, used only once, and disposed of after each use. The recovery and precision data demonstrated that our new approach, based on a combination of voltammetry with the SPCE sensor, provides reliable results. It is suggested that the simplicity of the developed methodology can form the basis for a rapid quality control procedure for the on-line analysis of gallic acid during wine production, using small, portable, commercial handheld instruments. Furthermore, this technology can be operated by personnel who do not necessarily have in-depth knowledge of electrochemical techniques, enabling small- to medium-sized local producers to test the quality of their wines on-site without the need for expensive and time-consuming techniques. The reported new method can be used as a platform for the development of novel, rapid point-of-test technology for determining a wide range of analytes during the quality control of alcoholic beverages.

## Figures and Tables

**Table 1:** The calibration equation, R2 values, and limits of detection (μM) were obtained using cyclic and differential-pulsed voltammetry with the plain SPCE sensor at pH 7.0.

Method	Equation	R^2^	STEYX	LOD (μM)	Slope
CV	y = 21.437x + 5.3253	0.9939	3.194	0.149	21.437
DPV	y = 0.2031x + 0.0493	0.9787	0.023	0.118	0.2031

**Table 2:** The original concentration of gallic acid found in banana wine using plain SPCE sensors with cyclic and differential-pulsed voltammetry.

BW	Original Concentration in BW (μM)	
	CV	DPV
1	7.113	7.647
2	7.165	6.891
3	7.434	6.671
4	7.571	8.602
5	7.561	8.037
**Average**	**7.369 ^a^**	**7.570 ^a^**
**SD**	**0.194**	**0.715**
**RSD (%)**	**2.60**	**9.40**

^a^ The same lowercase letters indicate no significant differences between methods.

**Table 3:** Recovery of gallic acid in fortified banana wine.

BW	Concentration Found in BW (μM)		GA Added (μM)	Recovery (Found—Added Concentration) in BW (μM)	
	**CV**	**DPV**		**CV**	**DPV**
1	14.529	14.669	7.500	7.029	7.169
2	15.065	15.157	7.500	7.565	7.657
3	14.241	14.942	7.500	6.741	7.442
4	14.357	14.786	7.500	6.857	7.286
5	14.713	15.196	7.500	7.213	7.696
**Recovery (%)**	-	-	-	**94.41**	**99.33**
**Average**	**14.581 ^a^**	**14.950 ^a^**	**7.500**	**7.081**	**7.450**
**SD**	**0.290**	**0.204**	**n/a**	**0.290**	**0.204**
**RSD (%)**	**2.00**	**1.40**	**n/a**	**4.10**	**2.70**

^a^ The same lowercase letters indicate no significant differences between methods. n/a: stands for not applicable.

**Table 4:** Identified studies employing various types of sensors to measure gallic acid in real samples.

Sensor Type	Technique	LOD	Linear Range	Sample Type	References
Plain SPCE sensor	CV Electrolyte: 10% ethanol—0.1 M PBS pH 7.0	0.149 μM	0.25 to 5.00 µM	BW	Our approach Foods, 2025
Plain SPCE sensor	DPV Electrolyte: 10% ethanol—0.1 M PBS pH 7.0	0.118 μM	0.25 to 5.00 µM	BW	Our approach Foods, 2025
Gold NPs decorated Kaolinite mineral modified SPCE	DPV Electrolyte: Britton-Robinson buffer pH 2.0	0.50 nM	0.002 µM to 40 µM	Black tea and pomegranate juice	[32]
Glassy carbon electrode modified with Co/FeOx@NC-800	DPV Electrolyte: 0.1 M PBS pH 3.0	1.30 nM	5.00 nM to 4.50 µM	Strawberries, grapes, tomatoes, and red wine	[22]
Cobalt oxide NPs-modified carbon-paste electrode	DPV Electrolyte: 0.1 M phosphate buffer pH 2.0	0.15 µM	0.10 µM to 1.00 µM	Red and white wine	[29]
Activated pencil lead electrode	DPV Electrolyte: 0.25 M phosphate-buffer solution pH 2.0	0.25 µM	0.49 to 24.30 µM	Black tea, green tea, and mango juice	[33]

## Data Availability

The original contributions presented in this study are included in the article. Further inquiries can be directed to the corresponding author.

## Abbreviations

The following abbreviations are used in this manuscript:

SPCE	Screen-printed Carbon Electrode
GA	Gallic Acid
BW	Banana Wine
CV	Cyclic Voltammetry
DPV	Differential-Pulsed Voltammetry
GC-MS	Gas Chromatography–Mass Spectrometry
HPLC	High-performance Liquid Chromatography
TLC FT-IR	Thin-layer Chromatography
DSC	Differential Scanning Calorimetry
TG	Thermogravimetry
LOD	Limit of Detection
STEYX	Standard Error of the Regression Line
